# Localisation using mini c-arm fluoroscopy of needles ingested by a woman with schizophrenia: a case report

**DOI:** 10.1186/1752-1947-3-6491

**Published:** 2009-03-18

**Authors:** Alper Parlakgumus, Sedat Yildirim, Naime Tokmak, Tamer Colakoglu, Kenan Caliskan, Ali Ezer, Gokhan Moray

**Affiliations:** 1 Baskent University, Department of General Surgery, Ankara, Turkey; 2 Baskent University, Department of Radiology, Ankara, Turkey

## Abstract

**Introduction:**

Our aim was to specify the use of mini C-arm fluoroscopy in a woman with schizophrenia who was suffering from abdominal pain because of ingested needles.

**Case presentation:**

Here we report the case of an 18-year-old Turkish woman with schizophrenia who was admitted to the emergency department with signs of an acute abdomen as a result of ingestion of multiple needles. This is the third case in the literature for which mini C-arm fluoroscopy has been used to localize metallic sewing needles.

**Conclusion:**

When intentional ingestion occurs, surgery is rarely required. It is hard to localize ingested sewing needles and mini C-arm fluoroscopy is a good alternative when metal detectors are not available for localization of metal sewing needles. We recommend this approach because it helps to avoid unnecessary exploration, shortens the duration of surgery and provides outstanding results.

## Introduction

Foreign body ingestion has been a fundamental subject in the area of pediatrics, emergency surgery and gastroenterology. Intentional ingestions of foreign bodies occur as a result of many factors such as self-demanding impulsivity, attention-seeking behavior in people with personality disorders, command hallucinations in the case of schizophrenia and in prisoners for the purpose of being transferred to a hospital. In the literature, mini C-arm fluoroscopy has rarely been rarely to detect metallic needles. Here we report an 18-year-old woman with schizophrenia who ingested multiple needles. This is the third case in the literature for which mini C-arm fluoroscopy has been used to localize metallic sewing needles.

## Case presentation

**Figure 1:**
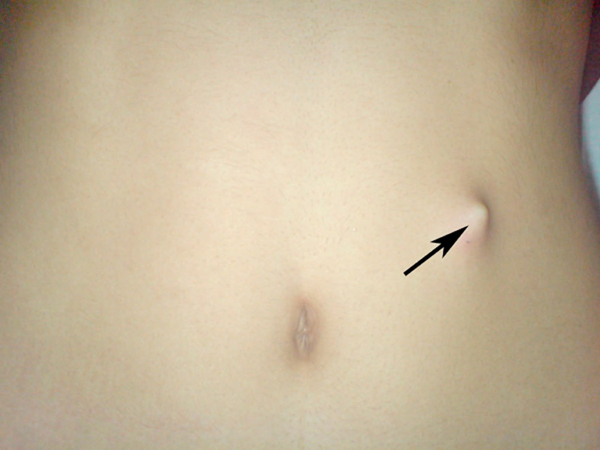
**Palpable prickling body under the skin on the left side of umbilicus**.

**Figure 2:**
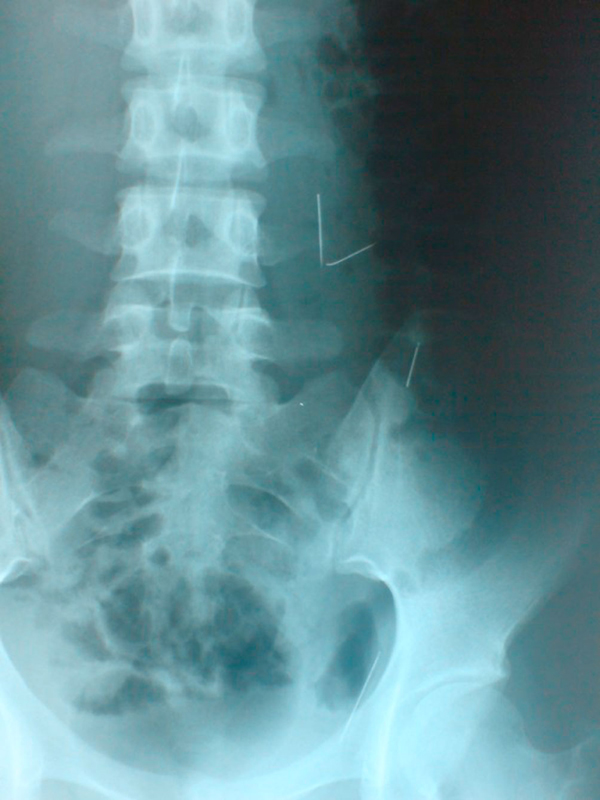
**Abdominal X -ray showing three pieces of needles**.

**Figure 3:**
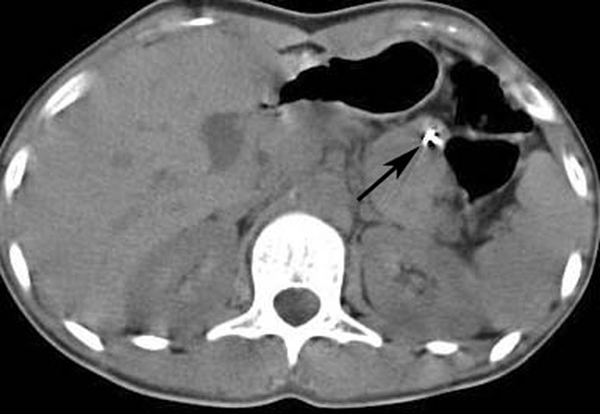
**Axial non-enhanced CT scan showed hyperdensity due to metallic sewing needles in the jejunal lumen**.

An 18-year-old Turkish woman with paranoid schizophrenia was admitted to the emergency department with a history of swallowing multiple sewing needles 20 days previously. She had progressive abdominal pain, nausea and vomiting. On physical examination, she was tachycardic, had abdominal tenderness, rigidity and a palpable prickling body under the skin on the left side of the umbilicus (Figure 1). Routine laboratory examinations revealed leucopenia and abdominal X-ray (Figure 2) and computed tomography (CT) showed three needles (Figure 3).

**Figure 4:**
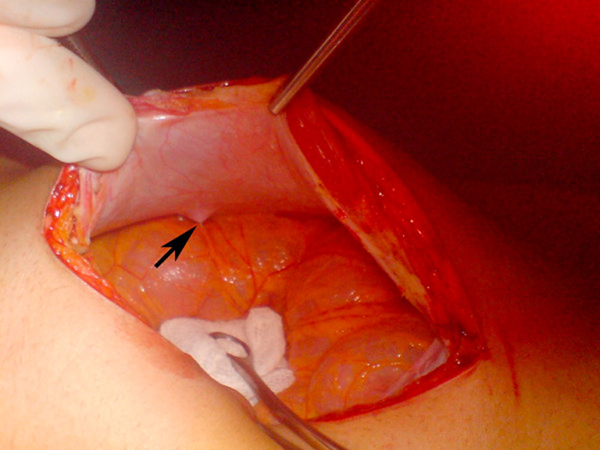
**One of the needles that migrated from the descending colon to the abdominal wall**.

**Figure 5:**
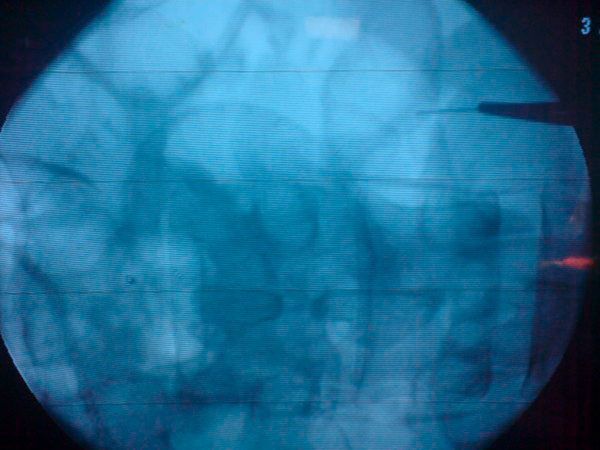
**The fluoroscopic view of one of the needles found in the jejunum**.

Abdominal exploration was performed and during the laparotomy, a needle that had migrated from the descending colon to the abdominal wall was found (Figure 4). In this case, we used mini C-arm fluoroscopy for identification of the needles and this approach obviated exploration and shortened the operation time. The other two needles were detected in proximal and distal parts of the jejunum with the aid of fluoroscopy (Figure 5). On the fifth postoperative day, she recovered fully and was discharged. Six months after the operation, the patient did not have any complaints. She was taking her psychiatric medications regularly, she was not losing weight and she was healthy and in good condition. X- Ray and CT did not show any swallowed foreign bodies at the follow-up examination.

## Discussion

Deliberate ingestion of foreign bodies should be kept in mind in patients with attempted suicide, attention-seeking behavior in personality disorders, self-damaging impulsivity and command hallucinations in schizophrenia when they present with abdominal pain to emergency departments. Another reason for this type of ingestion is encountered in prisoners or in cases of self-mutilation. Prisoners may deliberately swallow needles in order to be transferred from prison to a medical ward [1-3].

These data are important because ingestion of metal foreign bodies can be mistaken for ingestion of nonmetallic foreign objects ingested by a patient refusing to give a clear history of complaints. Occasionally this subset of patients is unconscious in the course of admission to hospital and foreign body ingestion must always be kept in mind. The patient in this case report presented to hospital 20 days after the event. In fact, the patient was not taken to the emergency service until her pain became intolerable. Fortunately, history and radiological examinations revealed the condition instantly.

The way to manage these ingested objects is also of great importance. It is suggested that if the foreign body stays in the cricopharyngeal sphincter or esophagus, endoscopic removal under sedation or anesthesia should be performed. The endoscopic procedure must be performed within 24 hours before the foreign bodies pass the upper gastrointestinal tract. Foreign bodies that stay for more than 24 hours cause an increased incidence of complications [4]. The American Society for Gastrointestinal Endoscopy suggested that only 10%-20% of foreign bodies may need to be removed endoscopically [5] and follow-up constitutes the major part of these cases. If the foreign body reaches the stomach, then the probability of this object passing through the gastrointestinal tract without causing any complications ranges between 80% and 90% and 1% of ingested objects will cause perforation [6-8]. An asymptomatic patient is a candidate for the follow-up approach.

Particular attention is needed in cases of sharp metallic bodies, small disk or button battery ingestion [5]. Sharp pointed objects located in the esophagus are a medical emergency. Objects located at or above the cricopharyngeus can be removed with direct laryngoscopy. If the location is inconclusive, rigid or flexible endoscopy can be used for objects located below this area. Most of the sharp pointed objects that enter the stomach will pass through the remaining GI tract without any problems; however, the risk of a complication due to a sharp pointed object can be as high as 35%. For this reason, if accomplished safely, endoscopy can help to retrieve sharp pointed objects that have passed into the stomach or proximal duodenum. Another alternative can be to make use of daily radiographs to document the passage of sharp pointed objects and to perform surgical intervention when the objects fail to progress for three consecutive days. Abdominal pain, vomiting, persistent temperature elevations, hematemesis and melena should be strictly observed and acted upon [5].

Small disk or battery ingestion may rapidly cause liquefaction necrosis and perforation particularly when a disk battery is in the esophagus. Radiography should be used to detect batteries located in the esophagus and they should be removed immediately to prevent fatal complications.

In the case presented here, abdominal examination and laboratory findings which showed a tendency to immunity degradation (leucopenia) helped to determine an operational approach. After a foreign body has perforated a viscus, it may lie in the lumen or adjacent to the perforation site or it may migrate to adjacent or distant organs or fall back into the lumen to perforate again or pass out without any other complications [9]. This case is interesting because the needle that migrated through the descending colon to the abdominal wall was lying perpendicular both to the lumen of the colon and abdominal wall, and was about to exit from the skin. This needle was the first finding noticed on physical examination.

It is hard to localize ingested sewing needles because they usually disappear in the digestive tract during manipulation and are impalpable manually. Metal detectors have been widely used in the localization of ingested metallic bodies but availability of these instruments is a great problem for many institutions [10].

In this case, we used mini C-arm fluoroscopy for identification of the needles and this approach obviated exploration and shortened the operation time.

The use of mini-C-arm fluoroscopy has become popular recently for several reasons. It provides quality images with the use of considerably less radiation than is used by a standard large C-arm. Radiation exposure with the standard C-arm fluoroscopy has been found to range from 1,200 to 4,000 mrem/min in selected orthopedic procedures. However, the use of a mini C-arm unit has been reported to cause radiation doses of 120 to 400 mrem /min. It is also easy to use and move from one place to another. In addition, the mini C-arm is less expensive to purchase and routine use does not require a radiology technician, which makes it very cost-effective [11].

This is the third case reported in the literature for which mini C-arm fluoroscopy was used to detect metallic foreign bodies [12]. Mini C-arm fluoroscopy can be a good alternative to metal detectors or standard large C-arm. However, further clinical trials are necessary to evaluate the feasibility of this device.

## Conclusion

Deliberate ingestion of foreign bodies should be kept in mind in patients with self-damaging impulsivity, command hallucinations in schizophrenia, attention seeking behavior in people with a personality disorder or prisoners who may want to be transferred to a hospital. When these occur, surgery is rarely required. It is hard to localize ingested sewing needles because they usually disappear in the digestive tract during manipulation and are impalpable manually.

Mini C-arm fluoroscopy is a good alternative when metal detectors are not available. We recommend this approach since it shortens operation time and avoids unnecessary exploration.

## Consent

Written informed consent was obtained from the patient and her family for publication of this case report and accompanying images. A copy of the written consent is available for review by the Editor-in-Chief of this journal.

## Competing interests

The authors declare that they have no competing interests.

## Authors' contributions

AP analyzed and interpreted the patient data, SY took part in the critical revision, NT interpreted the radiological images, KC took part in interpretation, TC took part in the surgical approach, AE drafted the article and GM took part in final approval of the manuscript.

All authors have made substantive intellectual contributions to this study and manuscript.
